# Effects of Global Warming on Predatory Bugs Supported by Data Across Geographic and Seasonal Climatic Gradients

**DOI:** 10.1371/journal.pone.0066622

**Published:** 2013-06-21

**Authors:** Tarryn Schuldiner-Harpaz, Moshe Coll

**Affiliations:** Department of Entomology, The Hebrew University of Jerusalem, Rehovot, Israel; The Pennsylvania State University, United States of America

## Abstract

Global warming may affect species abundance and distribution, as well as temperature-dependent morphometric traits. In this study, we first used historical data to document changes in *Orius* (Heteroptera: Anthocoridae) species assemblage and individual morphometric traits over the past seven decades in Israel. We then tested whether these changes could have been temperature driven by searching for similar patterns across seasonal and geographic climatic gradients in a present survey. The historical records indicated a shift in the relative abundance of dominant *Orius* species; the relative abundance of *O. albidipennis*, a desert-adapted species, increased while that of *O. laevigatus* decreased in recent decades by 6 and 10–15 folds, respectively. These shifts coincided with an overall increase of up to 2.1°C in mean daily temperatures over the last 25 years in Israel. Similar trends were found in contemporary data across two other climatic gradients, seasonal and geographic; *O. albidipennis* dominated *Orius* assemblages under warm conditions. Finally, specimens collected in the present survey were significantly smaller than those from the 1980’s, corresponding to significantly smaller individuals collected now during warmer than colder seasons. Taken together, results provide strong support to the hypothesis that temperature is the most likely driver of the observed shifts in species composition and body sizes because (1) historical changes in both species assemblage and body size were associated with rising temperatures in the study region over the last few decades; and (2) similar changes were observed as a result of contemporary drivers that are associated with temperature.

## Introduction

Changes in global climate over the past decades are well documented; average global surface temperature, for example, has risen by approximately 0.75°C during the past 100 years (1906–2005) [1]. Most of the warming during this period occurred in the last 50 years: average temperatures rose by approximately 0.13°C per decade between 1956 and 2005 [1]. The extent of the temperature increase, however, varied among regions. For example, summer warming trends in most of the Mediterranean basin were found to greatly exceed global trends [2].

The ecological impacts of climate change have been demonstrated for a wide range of organisms, at both individual and population levels [3], [4]. In particular, evidence is accumulating on climate-driven changes in species abundance and distribution; the distribution range limits of a wide variety of taxa have shifted, both in latitude and altitude, in accordance with predicted effects of global warming [4], [5], [6]. Furthermore, climate is known to affect morphological traits such as body size and related allometric characteristics [7], [8], [9]. In turn, body size has numerous dramatic effects on animal fitness traits [10], [11], [12].

Insects may be especially sensitive to climatic fluctuations, because as small ectotherms, their physiological and life history characteristics are greatly affected by variation in temperature and humidity [3], [13]. Yet, insects are able to respond rapidly to climate changes and adapt to the changing environment due to high reproductive potential and relatively short generation time [14]. In addition, a high degree of mobility through flight allows insects to rapidly shift or expand their geographic ranges to previously unsuitable areas. The effect of global warming on the abundance, distribution and range limits of insects has in fact received much attention: range shifts have been well documented in Lepidoptera [6], [15], [16] and to a lesser degree in several other arthropod orders [13], [14], [16]. Yet effects of climate change on insect morphology have been largely neglected.

In the present study, we investigated the effect of climate warming on (i) the species assemblage and (ii) individual morphometric traits of sympatric *Orius* species (Heteroptera: Anthocoridae). First, we examined historical records for long-term changes in species composition and bug morphology. We then tested whether similar changes in species distribution and morphometric traits could be detected now across seasonal and geographical climatic gradients. Finding (i) coordinated climate related changes at two separate levels: species composition and individual morphometric traits, and (ii) similar effects of warming across historical, geographical and seasonal dimensions would strongly support our hypothesis that historical climate warming is the main driver to changes in *Orius* species over the past decades.

Israel is especially suitable for this study because of its location in a transition zone between a southern desert climate and a northern sub-humid Mediterranean climate [17]. In addition, Israel has experienced a high level of warming over the past few decades [18], which on average, exceeded global trends. As a result, we would expect the effect of climate warming to be more pronounced in this region.

The genus *Orius* includes approximately 90 species worldwide [19] (TSH & MC, unpublished data). These bugs are mostly generalist predators commonly found in flowers of herbaceous vegetation; several species are used in biological control programs to suppress agricultural pests [20], [21]. The geographic ranges of the three most abundant *Orius* species in Israel [22] overlap in the Mediterranean basin. *O. albidipennis* is a desert-adapted species that is abundant mainly from the Mediterranean basin southwards. In contrast, *O. laevigatus* and *O. niger* are temperate species; the former is found principally in the Mediterranean basin and the latter from the Mediterranean basin northwards [23]. In accordance with their range of distribution, the three species differ in optimal temperature range for development, reproduction and survival. For example, reproduction and development of *O. laevigatus* could be maintained at lower temperatures than those of *O. albidipennis* (lower development threshold of ∼ 11°C and ∼ 14°C, respectively). Moreover, the fecundity of *O. laevigatus* at high temperatures, such as 35°C, is reduced to a greater extent than that of *O. albidipennis* [24], [25]. Finally, it seems that *O. niger* is well adapted to a wider range of climatic conditions [26], [27].

The main motivation for this study was therefore the strong climatic gradient found across relatively short distances in Israel, the distribution patterns of *Orius* species in the region and their economic importance as natural enemies of agricultural pests.

## Materials and Methods

### 1. Ethics Statement

This study did not involve endangered or protected species. Field collection sites were not located in nature reserves and did not include protected or privately owned land. Therefore, no specific permissions were required for collecting the study insects and for field collection locations.

### 2. Data Collection

To determine whether the relative abundance of *Orius* species in Israel has changed over time, data from present and historical *Orius* collections in Israel were compiled based on: (i) specimens deposited in the National Entomological Collection (Tel Aviv University) and in the Plant Protection and Inspection Services (PPIS) collection in Beit Dagan (permission was granted to access both collections and loan specimens to be examined where they are housed); (ii) two published reports on *Orius* collections in Israel [28], [29]; (iii) collections made during 2001–2 for studies by Groenteman (2004) [30] and Shouster (2003) [31] and (iv) our 2009–10 survey. Overall, present and historical records included approximately 4,300 *Orius* specimens collected in Israel over a period of seven decades (1942–2010).

To control for biases due to variation in sampling time and site in different collections, we also compared specimens collected by R. Linnavuori [28], an expert taxonomist of Anthocoridae, from the central coastal plain during the summer of 1958 to specimens collected in the present survey during the same season in the same area.

Historical changes in the relative abundance of *Orius* species were then examined with regard to climate warming in Israel over time. Trends in daily mean temperature were determined by analysis of climatic data obtained from the European Climate Assessment & Dataset website (http://eca.knmi.nl). Four meteorological stations were chosen to represent different climatic regions in Israel: Mt. Kna'an (mesic-Mediterranean climate in the Galilee), Beit Dagan (dry-Mediterranean of central coastal plain), Nahal Hazerim (arid Negev desert) and Eilat (extremely arid southern Arava valley). Available weather data span the years 1965–2010 for all except Nahal Hazerim station, from which data were available only for the years 1967–2003.

### 3. Field Collections

**Figure 1:**
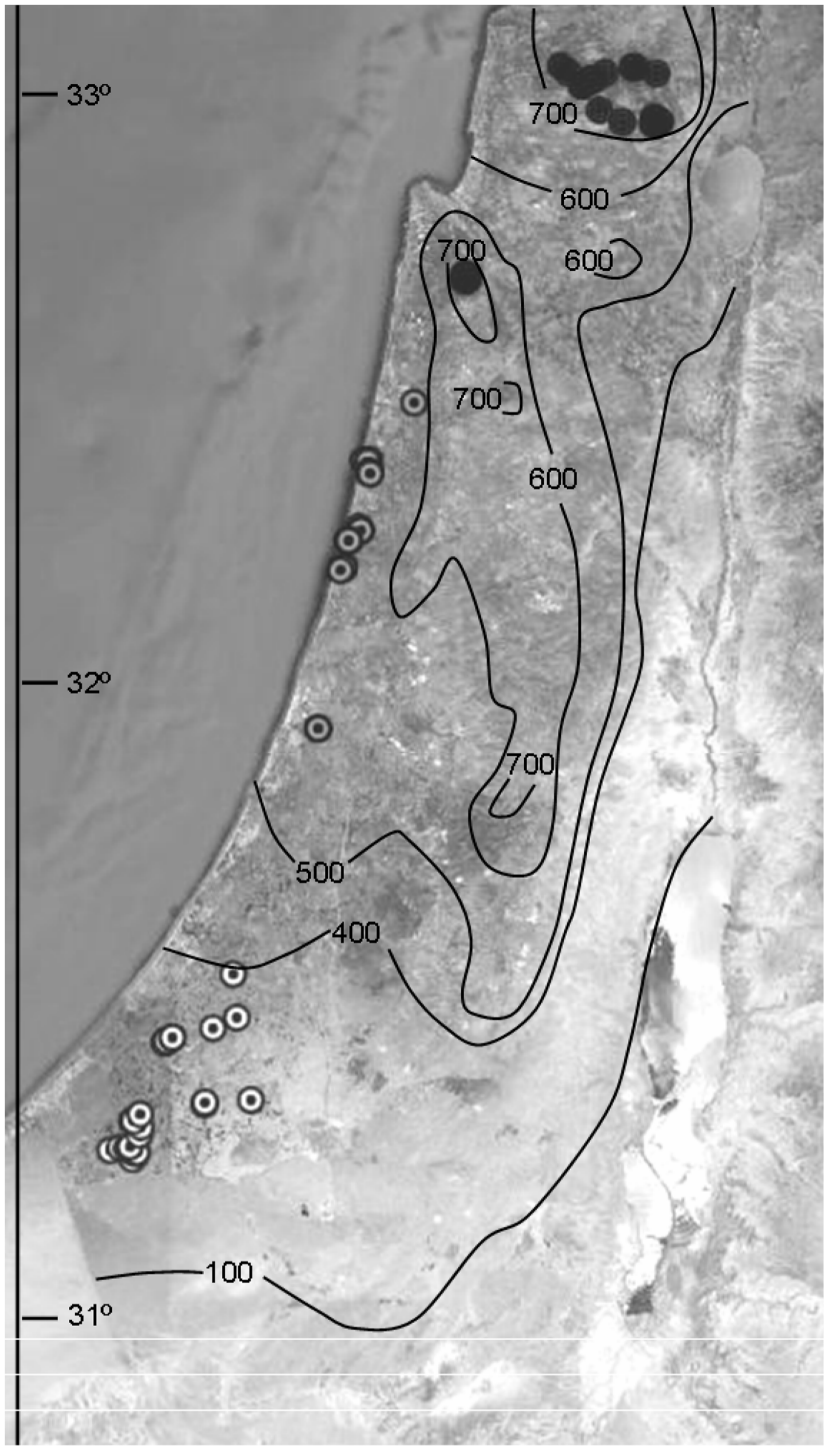
Field collection sites (2009–10) at three regions along a north to south climatic gradient in Israel (Google earth) (Mountainous Mediterranean - dark grey marks, Mediterranean plain - light grey marks, and Semi-arid - white marks), superimposed over mean annual precipitation isoclines (in mm; Goldreich 2003).

Approximately 2,500 *Orius* specimens were obtained in 109 field collections during 2009–2010 to examine changes in the relative abundance of *Orius* species along climatic gradients in Israel. The collections were carried out during four different seasons (January, April, August and November), and collection sites were divided according to three distinct regions along a north to south climatic gradient (Fig. 1). The northernmost region, termed ‘Mountainous Mediterranean’, included Mt. Carmel and Mt. Meron (over 400 m ASL), which are characterized by Mediterranean maquis vegetation, a mean annual temperature of 15–19°C and annual precipitation exceeding 700 mm. The ‘Mediterranean plain’ region encompassed the coastal plain area, which is characterized by Mediterranean garrigue vegetation, a mean annual temperature of 17–21°C and annual precipitation of 500–700 mm. The southernmost region in our survey, which was termed ‘Semi-arid’, included the Western Negev, a dry area characterized by a mean annual temperature of 19–21°C and annual precipitation of 100–400 mm [17]. Bugs were collected at five to nine different sites in each region, depending on flower availability. The same sites were re-visited every collection season, unless flowers became unavailable. The most northern collecting sites are about 200 km from the most southern ones.

Two to four hundred flowers were collected at each site (ca. 10 m^2^), placed in paper bags and kept cool during transfer to the laboratory. In the lab, bugs were extracted from the flowers by shaking. Species of collected flowers varied according to season and location; they included mostly Asteraceae species such as *Verbesina encelioides*, *Helianthus annuus* and *Chrysanthemum coronarium*, but also non-composites such as *Solanum elaeagnifolium* (Solanaceae). To avoid possible bias due to collection of bugs from different plant species, the relative abundances of *Orius* species were compared among plant species that flower simultaneously in the same area. No significant differences were found among flower species, except for higher relative abundance of *O. laevigatus* on *Verbesina encelioides* compared to *Chrysanthemum coronarium* in the Mediterranean plain region, during April (constituting 11% and 1% of the bugs on these plants, respectively *χ* ^2^_2_ = 15.9, *P*<0.001). Yet this difference is probably due to the small number of *O. laevigatus* bugs that were collected during this month from both flower species (22 bugs in total).

After extraction from the flowers, specimens were identified to subgenus by the presence or absence of a micro-seta in each corner of the pronotum (following Péricart 1972 [23]). Further identification to species was conducted separately for each sex. Male specimens were identified by examining their genitalia [23], [32]. For genitalia preparation, specimens were first soaked in a KOH solution (10%) for 24 hours. The genitalia were then extracted using a minuten pin (following Ferragut and González-Zamora 1994 [32]). Female specimens were identified according the opercula shape of eggs they deposited (following Schuldiner-Harpaz and Coll 2012 [33]). After identification, the relative abundance of each species was calculated separately for each of the three climatic regions and four seasons. Due to differences in sex ratio among the species (0.11, 0.33 and 0.38 males to one female for *O. laevigatus*, *O. niger* and *O. albidipennis*, respectively), relative abundance of the bugs in each region and month was calculated separately for each sex.

### 4. Morphometric Measurement

Field collected females from the present survey were measured for thorax width and wing length (the males were excluded to control for sex-related variation in body size). Thorax width was defined as the width of the widest part of the pronotum. Wing length was measured in a straight line from the axillary area, at the base of the right hemelytron (forewing), to the wing apex. To carry out this measurement, the wing was detached from the body and placed on a slide covered with double-sided transparent tape. To minimize measurement error, all specimens were measured by the same person and only after all of the collections had been done. Both thorax width and wing length were measured to the nearest 0.015 mm using an eyepiece graticule under a stereoscope at 32 x magnification. Measurement error was estimated based on a repeated measurement of 30 specimens. A high correlation was found between repeated measurements (r = 0.93 and r = 0.99 for thorax width and wing length, respectively). For each *Orius* species, wing length, thorax width, and the ratio between them were compared among collection regions and seasons.

Morphometric measurements of historically-collected specimens were taken only for females collected during the 1980’s, for which specimens were available and in good condition. Because of variation in wing positioning, wing length could not be recorded accurately in a non-destructive manner (i.e., without detaching the wings) for all historically-collected specimens.

### 5. Statistical Analysis

JMP 7 [34] was used for all statistical analyses. Trends in daily mean temperature were analyzed using linear regressions. Species relative abundances were compared among years using a two-way log-likelihood ratio test. The relative abundance of *Orius* species collected in the present survey was compared among collection months and regions, using a three-way log-likelihood ratio test (with species, season and region as main effects). Bug samples were pooled across all sites within the same region and month. For all but 5 of the 109 collection samples, relative species abundance was found to be homogeneous between different samples within the same region and month. Excluding these five sites did not change the results significantly, and they were retained in the analyses presented here. Only males were identified to species in 2009 whereas both sexes were identified in 2010. Male data were pooled over the two years because they showed a similar trend.

Thorax widths of specimens from historical collections and from the present survey were compared between years, for each season separately, using one-way ANOVA. Morphometric traits of specimens collected in the present survey were compared among collection months and regions using a two-way ANOVA. Means within ANOVA were compared using Tukey’s HSD test. Data that did not meet ANOVA assumptions were either log-transformed or analyzed using non-parametric tests (Mann-Whitney and Kruskal-Wallis).

## Results

### 1. Long-term Trends in Daily Mean Temperature

**Figure 2:**
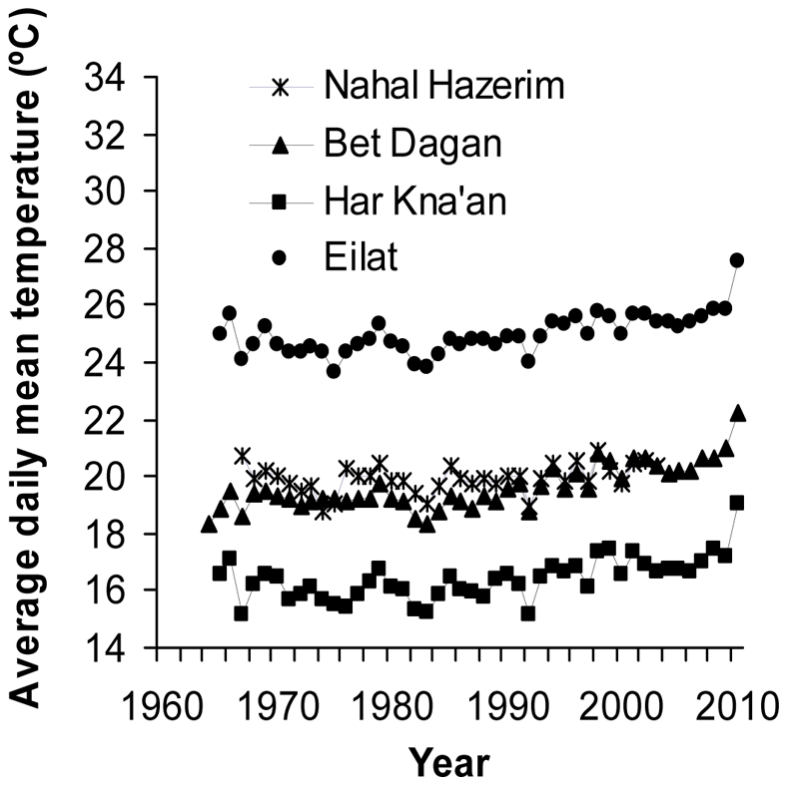
Daily mean temperature (°C) recorded over a period of five decades in four meteorological stations representing different climatic regions in Israel: Mt. Kna’an (mesic-Mediterranean climate in the Galilee), Beit Dagan (dry-Mediterranean of central coastal plain), Nahal Hazerim (arid Negev desert) and Eilat (extremely arid southern Arava valley). Data from the European Climate Assessment & Dataset (http://eca.knmi.nl).

Temperatures at all four stations have risen since the 1960’s, although it was significantly so (p<0.0001; 0.3–0.4°C per decade) only in three of the stations (those for which data are available also after 2003). Further examination showed that the increase in daily mean temperature occurred mainly from the mid 1980’s onwards. Changes in temperature before 1985 were not significant in any of the stations. Yet over the last 25 years, daily mean temperatures have increased by 1.5–2.1°C in Har Kna’an, Bet Dagan and Eilat, and by 0.5°C in Nahal Hazerim (Fig. 2). These changes were highly significant at the first three stations (*P*≤0.0001) and marginally not significant at the latter station (*P* = 0.08; probably because it did not include data for 2004–2010).

### 2. Species Relative Abundance

#### 2.1. Historical data compared to the present

**Figure 3:**
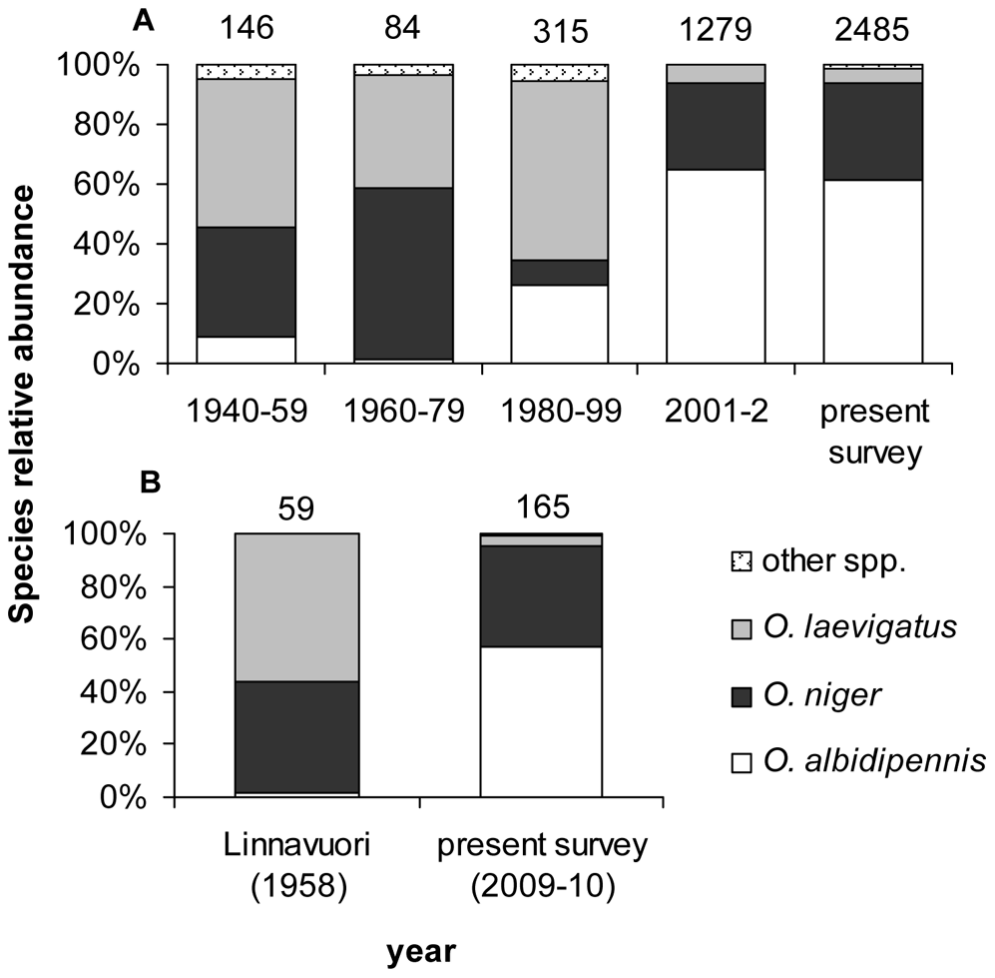
Changes in the relative abundance (%) of *Orius* species over time. (A) Comparison of all specimens collected during five periods. (B) Comparison of specimens collected and identified by R. Linnavuori during June-August of 1958 to specimens collected during the same months in the same area in 2009–10. Number of specimens is indicated above each column.

The three most abundant *Orius* species found both in historical collections and in the present survey were *Orius niger*, *O. albidipennis* and *O. laevigatus* (Fig. 3). Other, much less abundant species included: *O. horvathi* (which constituted 2% of *Orius* bugs in the present survey), *O. laticollis*, *O. minutus*, *O. canariensis* and one specimen of *O. majusculus*, which together constituted between 4–6% of all historical *Orius* specimens. In addition, *O. pallidicornis* was found in small numbers in historical data; almost all specimens were collected on *Ecbalium elaterium*, which is its main food plant [35]. As a result, it is likely that focused collections on *Ecbalium elaterium* were carried out with the sole purpose of finding this species. To avoid possible bias, *O. pallidicornis* was therefore not included in the analysis.

Comparison of the relative abundance of *Orius* species revealed significant differences among years (*G*_12_ = 1060.2, *P*<0.0001). The relative abundance of *O. laevigatus* has decreased from 50%, 38% and 60% during 1940–59, 1960–79 and 1980–99, respectively, to 4–6% during 2001–2 and the present survey. In contrast, the relative abundance of *O. albidipennis* has increased gradually from 9% and 1% during 1940–59 and 1960–79, respectively, to 26% during 1980–99 and 65% and 62% in 2001–2 and in the present survey, respectively. No clear trend was recorded in the relative abundance of *O. niger* over these years (Fig. 3a).

The controlled comparison between specimens collected by R. Linnavuori in 1958 to those collected at the same season and area (during the summer in the central coastal plain) in the present survey showed similar changes to those found in the whole data set; highly significant differences were found in species relative abundance between the two sets of specimens (*G*_2_ = 104.5, *P*<0.001). The relative abundance of *O. laevigatus* in that region, in the summer, decreased from 56% in 1958 to 4% in 2010. Conversely, *O. albidipennis* increased in relative abundance from 2% in 1958 to 57% in 2010 (Fig. 3b).

#### 2.2. Changes along climatic gradients

**Figure 4:**
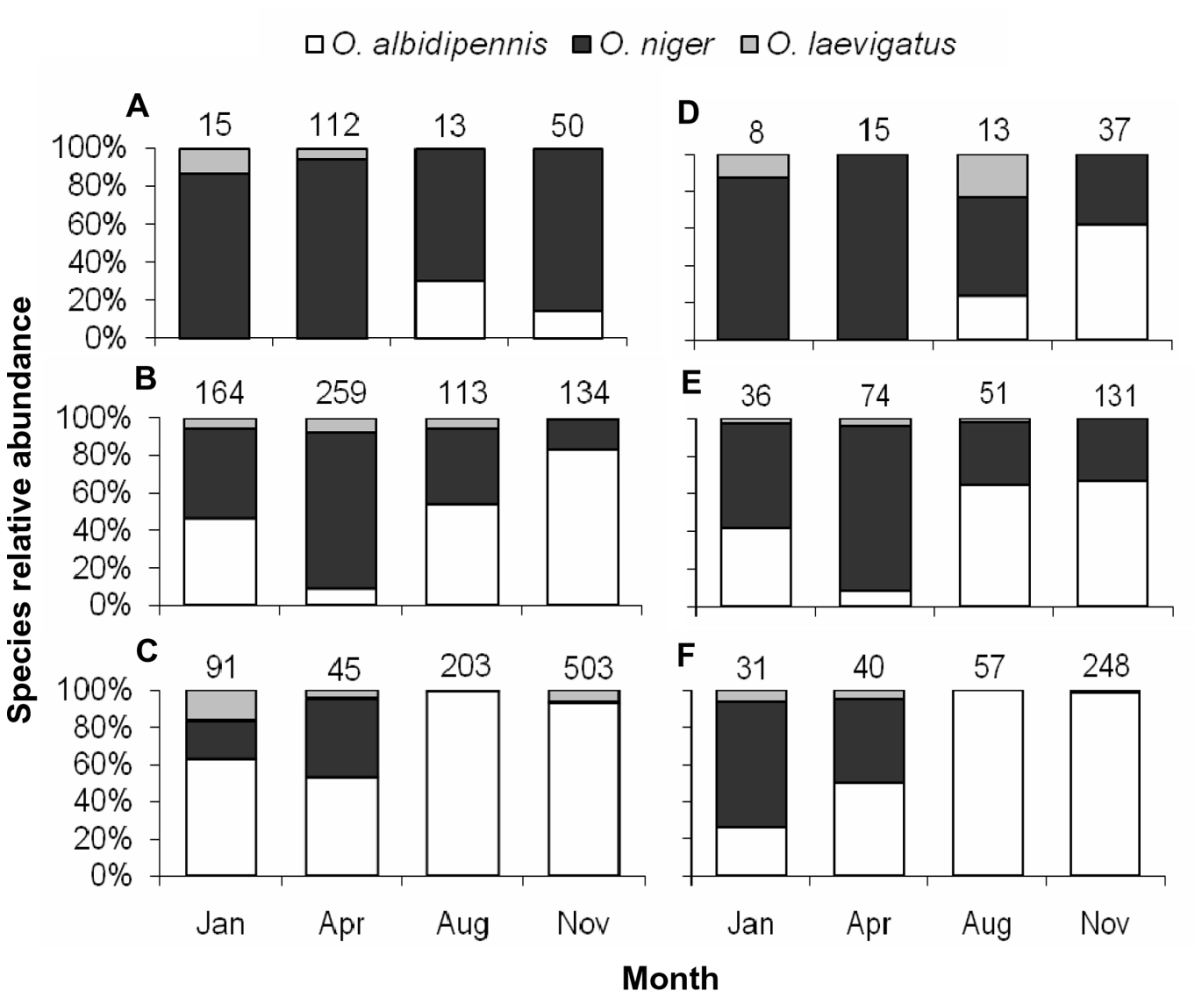
Relative abundance (%) of three *Orius* species based on female specimens (A, B, C) collected in 2010 and on male specimens (D, E, F) collected in January 2010 and in April, August and November 2009–10. Collections were carried out during four seasons in three regions: Mountainous Mediterranean (A, D), Mediterranean plain (B, E) and Semi-arid desert (C, F). Number of specimens is indicated above each column.

Results of a three-way log-likelihood ratio test detected significant interactive effects between the region and month of collection on species composition (females: *G*_12_ = 96.3, *P*<0.0001; males: *G*_12_ = 55.3, *P*<0.0001). However, changes in species relative abundance throughout the year were similar in all regions (Fig. 4); for both males and females, the relative abundance of *O. albidipennis* was highest during August and November, while the relative abundance of *O. niger* was highest during January and April (significant effect of collection month: *G*_6_ = 163.1, *P*<0.0001 and *G*_6_ = 174.7, *P*<0.0001, respectively for females and males; Fig. 4). In addition, in all collection months, the relative abundance of *O. albidipennis* increased from north to south, whereas the relative abundance of *O. niger* increased from south to north (significant effect of the collection region: *G*_4_ = 328.3, *P*<0.0001 and *G*_4_ = 91.4, *P*<0.0001, respectively, for females and males; Fig. 4).

### 3. Morphometric Characteristics

#### 3.1. Historical data compared to the present

**Figure 5:**
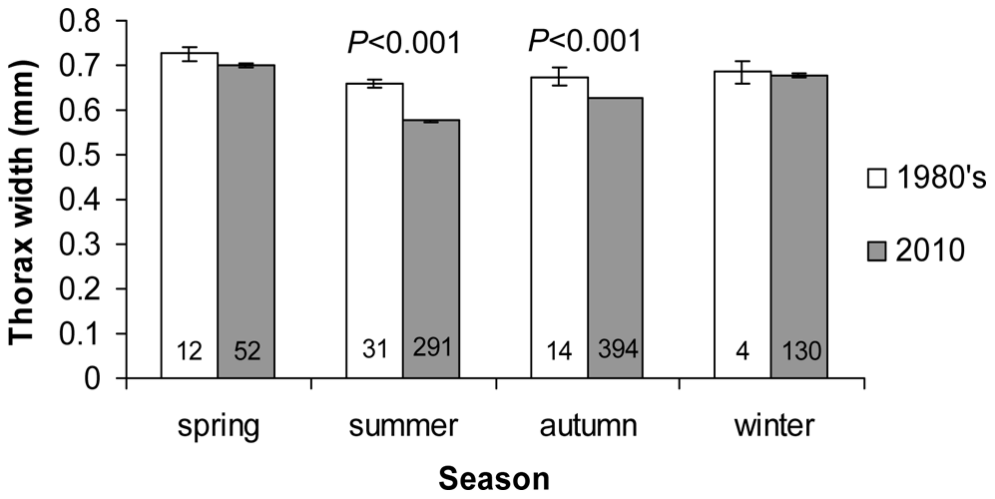
Thorax width (mm±se) of *Orius albidipennis* specimens collected during four seasons in the 1980’s compared to those collected in a 2009–10 survey. Sample size is indicated for each bar.

Taken across all seasons, thorax width of *O. albidipennis* was significantly larger in specimens collected in the 1980’s (0.68±0.01 mm) than in those from the present collections (0.62±0.01 mm; *F*_1,926_ = 56.5, *P*<0.0001). This difference was significant only in the summer and autumn (Fig. 5). Likewise, thorax width of *O. laevigatus* was significantly larger in specimens collected in spring in the 1980’s (0.79±0.01 mm) than in specimens collected during the same season in the present survey (0.73±0.01 mm; *F*_3,_ = 16.4, *P*<0.0001). Small sample sizes did not allow the comparison of *O. laevigatus* thorax width in other seasons and for *O. niger* among years.

#### 3.2. Changes along climatic gradients

For both *O. albidipennis* and *O. niger*, the abundant species in the present survey for which samples sizes were sufficient for proper analysis, significant interactive effects of collection region and season on bug morphometry were detected (table 1). However, wing length, thorax width and wing to thorax size ratio all showed similar trends throughout the year in all regions. Average wing length was 25% larger in January than in August, in both species; average thorax width was 18% and 21% larger in January than in August for *O. albidipennis* and *O. niger*, respectively; and average wing to thorax size ratio was 6% and 3% higher in January than in August for *O*. *albidipennis* and *O. niger*, respectively (see Table S1 for detailed values).

**Table 1:** ANOVA tables^1^ for the effect of collection month and region on three morphometric traits of *Orius albidipennis* and *O. niger*.

		Wing length (mm)			Thorax width (mm)			Wing/thorax size ratio		
***Orius albidipennis***		(*n* = 829)			(*n* = 852)			(*n* = 829)		
		*df*	*F*	*P*	*df*	*U*	*p*	*df*	*F*	*P*
	Month	3	481.9	**<0.001**	3	44 3.2	**<0.001**	3	157.6	**<0.001**
	Region^2^	1	1.7	0.2	1	35.3	**<0.001**	1	0.1	0.8
	Month×region	3	18.3	**<0.001**				3	7.3	**<0.001**
***Orius niger***		(*n* = 548)			(*n* = 567)			(*n* = 548)		
		*df*	*F*	*p*	*df*	*F*	*p*	*df*	*H*	*P*
	Month	3	71.9	**<0.001**	3	42.1	**<0.001**	3	44.9	**<0.001**
	Region	2	0.2	0.8	2	0.5	0.6	2	2.3	0.3
	Month×region	6	2.4	**0.03**	6	3.1	**0.006**			

1 Two-way ANOVA was used in all analyses except *O. albidipennis* thorax width and *O. niger* wing/thorax size ratio, which were analyzed using Mann-Whitney and Kruskal-Wallis tests, respectively, and therefore do not include interactions. P-values lower than 0.05 are indicated in boldface numerals.

2 Due to small sample size, analysis for *O. albidipennis* does not include data from the Mountainous Mediterranean region.

Results do not support the idea that seasonal variation in body size is related to changes in population density. Regression analyses revealed non-significant associations of pronotum width with bug density (number of individuals per flower). For example, thorax width of *O. albidipennis* was not correlated significantly with its population density or with that of all *Orius* bugs (*R^2^* = 0.01, *p* = 0.3 and *R^2^* = 0.001, *p* = 0.9, respectively).

Collection region had no significant effect on any recorded morphometric traits, apart from thorax width of *O. albidipennis* (non-parametric analysis, table 1), which was larger in specimens from the Mediterranean plain (0.63±0.003 mm) than in those from the Semi-arid region (0.61±0.002 mm).

## Discussion

Results of our study strongly support the hypothesis that climatic warming over the last few decades altered the composition of *Orius* species and their morphometric traits in the eastern part of the Mediterranean basin.

### 1. Long-term Trends in Daily Mean Temperature

The increase in daily mean temperatures that was detected in the present study is consistent with similar trends found by Shahami & Morin [18] in minimum and maximum temperatures in a more extensive study throughout Israel. Both studies indicate that over the past five decades, temperature increases in Israel greatly exceeded average global trends. Importantly, we found that most of the increase in temperature in Israel was recorded since the mid 1980’s (overall increase of between 0.5–2.1°C at different sites).

### 2. Species Relative Abundance

The recorded major shifts in the relative abundance of *Orius* species in Israel over the past seven decades parallel regional warming trends during that time. The relative abundance of the desert-adapted species *O. albidipennis* was considerably higher in the present survey and in surveys conducted in 2001–2 than in the older historical records taken before significant warming occurred (data older than 30 years or so). In contrast, the once prevalent northern species *O. laevigatus* constituted only a minor part of the *Orius* species complex in the present survey and in that of 2001–2. This decline in the relative abundance of *O. laevigatus* is particularly surprising, because since 1994 this species has been mass-produced and released in large numbers in agricultural crops such as sweet pepper, to biologically control pests (S. Steinberg, personal communication). These released populations might be expected to spill over and become established in natural habitats. Yet instead of detecting an increase in the relative abundance of *O. laevigatus* over time, this species was relatively rare in both the 2001–2 and present surveys.

No clear trend was observed in the relative abundance of *O. niger* over the years. This species appears to be less affected by regional historical warming. This may be because it is better adapted to a wide range of habitat and temperature conditions [26], [27] and is widely distributed throughout the Palearctic region [23].

Strong geographical and seasonal changes in relative abundance of *Orius* species in the present survey correspond to similar changes in *Orius* species abundance recorded in Spain and Italy [20], [36], [37]. Our results indicate a clear shift in dominance; the relative abundance of *O. niger* was highest in the northern region and during winter, with a carry-over effect into spring. Dominance shifted towards *O. albidipennis* in the southern, semi-arid region, and in summer, with a carry-over effect into autumn. A survey conducted by Chyzik and Ucko [38] in the hyper-arid Arava valley in Israel (with temperatures acceding 40°C on more than 40 days each year, a relative humidity of less than 20% and 25 mm average annual rainfall), revealed that *O. albidipennis* comprises almost 95% of the *Orius* species collected in this region throughout the year.

Our hypothesis, that the pronounced rise in the relative abundance of *O. albidipennis* over the past several decades was driven by climate warming, is therefore supported by two independent lines of evidence: the increase in *O. albidipennis* relative abundance (i) during warmer seasons and (ii) along a north to south climatic gradient. Moreover, the seasonal shift in dominance between *O. albidipennis* and *O. laevigatus* found by Lacasa *et al.* [36] in southeastern Spain provides further support to the notion that the shift in species dominance over the years in Israel is climate driven.

The historical changes observed in the relative abundance of *Orius* species in Israel may reflect shifting range limits on a larger, more global scale. The overlap between the northern range limit of *O. albidipennis* and the southern range limit of *O. laevigatus* may be shifting northward within the Mediterranean basin, resulting in an increase in the relative abundance of *O. albidipennis* and a decrease in the relative abundance of *O. laevigatus* in transition zones, such as Israel. Similar changes in range have been reported in butterfly and dragonfly species that were once confined to Africa and have now moved northwards into the Mediterranean basin [14], [39]. Further surveys in more northern areas are needed to assess whether *O. laevigatus* distribution has shifted northward or has shrunk due to global warming.

Conclusions concerning the influence of climate change on shifts in species abundance should be made with caution. Other factors, such as changes in land use and vegetation composition over time, may have also been involved. These changes may have altered species abundance directly or indirectly by changing food availability and physical habitat properties. Yet the central role climate warming has in altering *Orius* species composition is strongly supported by the fact that (i) similar changes were recorded both between seasons and across a geographic climatic gradient and (ii) similar compositions of *Orius* species were found on different plant flowers.

### 3. Morphometric Characteristics

Body size, evaluated by thorax width and wing length, differed significantly for bugs collected in different seasons in the present survey. Both thorax width and wing length were largest during the cold season and smallest during the warm season. Variations in body size along geographic gradients within Israel have been found in other insect species [40], [41]. In the present study however, *O. albidipennis* thorax width was the only parameter that varied significantly among collection regions. Yet, differences among regions were much smaller compared to the recorded differences among seasons and are therefore not likely to have much biological significance (0.02 mm difference between thorax width in the Semi-arid and Mediterranean plain regions, compared to 0.13 mm difference between January and August). Interestingly, the difference in mean temperature among seasons was higher than that among the geographic regions used in this study (differences in the magnitude of 10–20°C and 0.5–10°C, respectively). This may underlie the in-significant variation found in morphometric traits across collection regions in our study.

Body size is one of the most ecologically-important morphological traits [10], [12]. It has pronounced effects on animal physiology, longevity, reproduction, competitiveness and many other fitness components [10], [11], [12] and related ecological processes [42]. It is therefore important to understand how environmental factors produce changes in body size. Bergmann’s rule predicts that endotherms from higher latitudes and altitudes should be larger than those from warmer climates [43]. This phenomenon has been reported in ectotherms as well [11], when several insect taxa were compared both intra- and inter-specifically [8], [44]. Indeed, *O. albidipennis*, *O. niger*, and possibly other *Orius* species, may follow Bergmann’s rule and attain larger bodies in colder climates. Blanckenhorn and Demont (2004) suggested that small arthropods with short development times show Bergmann clines, while large univoltine arthropods are likely to experience season duration constraints and thus display converse Bergmann clines. Our findings support this proposition; *Orius* bugs, which exhibited Bergmann clines, are multivoltine and their development is not limited by season length.

The recorded seasonal variation in thorax width in the present study corresponded nicely to historical changes in thorax width. Contemporary bugs had smaller thorax widths during the warm seasons and likewise, present collections yielded bugs with a smaller average thorax width compared to conspecifics from the 1980’s. In addition, the most pronounced decline in *O. albidipennis* thorax width was detected during the summer, when historical warming was most evident in Israel [18]. To date, reduction in body size over time due to global warming has been observed mainly in birds [45]. Our results suggest that this phenomenon may be occurring in insects as well.

In addition to thorax width and wing length, wing to thorax size ratio in the present survey was also larger in cold than warm seasons. The ratio between wing size and body size, or body mass, is an indicator of flight ability [7], [46] and has been shown to vary under different thermal conditions in other insect species [7], [41], [46], [47]. The development of larger wings in proportion to body size or weight has been suggested as a mechanism to overcome lower wing beat frequency in cold compared to warm environments [7], [46]. If so, the influence of climatic warming on flight ability may affect animal demography both through its effect on traits associated with body size, and by shifting the dispersal-oogenesis trade-off [48]. Interestingly, these two effects are expected to have an opposite influence on population growth rate under warmer conditions; the former effect by producing less fecund females with smaller body sizes, and the latter through greater allocation of resources to egg production instead of the development of larger wings.

### 4. Implications for the use of *Orius* Species in Biological Control

Biological control of insect pests is often achieved by relying on naturally-occurring predators in the field. For example, pest populations of the western flower thrips are suppressed by *Orius* species that appear spontaneously in insecticide-free pepper and strawberry fields in Israel [38], [49]. Because different *Orius* species vary in their ability to control insect pests, the effect of global warming on species composition may affect the outcome of biological pest control programs that rely on naturally-occurring *Orius* species.

In addition, morphometric traits of *Orius* bugs may also influence their efficacy as natural enemies of agricultural pests. Larger predators are able to forage over larger areas and to better subdue their prey than are smaller ones. Also, body size often affects predator competitiveness [11] and therefore the likelihood of surviving intraguild predatory attacks. Finally, body size affects longevity and reproduction [11], [42], which in turn may determine the ability of natural enemies to become established in the field and suppress pest populations.

### 5. Conclusions

A dramatic shift in *Orius* species dominance has taken place over the last 25 years or so; the Mediterranean *O. laevigatus* species has become rare, while *O. albidipennis*, a southern, desert-adapted species, has increased considerably in relative abundance. In addition, contemporary specimens of the latter species were smaller than those collected in the 1980’s. These changes co-occurred with rising temperatures in Israel over the past 25 years. That climate warming may be involved in these changes at the community and individual levels of *Orius* bugs is strongly supported by similar changes over two other, separate climatic gradients in Israel: (i) seasonal and (ii) geographical. This shift in *Orius* species composition in Israel may be the result of a northward expansion or shift in *Orius* species distribution in the region brought about by global warming.

## Supporting Information

Table S1 **Average measurement of morphometric traits, across seasonal and geographic climatic gradients, in specimens from the present survey.** (a) *Orius albidipennis* and (b) *O. niger*.
(DOC)
