# Supplementary material for: Effects of Global Warming on Predatory Bugs Supported by Data Across Geographic and Seasonal Climatic Gradients
Source: PLoS One. 2013 Jun 21;8(6):e66622. doi: 10.1371/journal.pone.0066622 (PMC3689781; doi:10.1371/journal.pone.0066622)
Supplement: Table S1 — Average measurement of morphometric traits, across seasonal and geographic climatic gradients, in specimens from the present survey. (a) Orius albidipennis and (b) O. niger. (DOC) [file pone.0066622.s001.doc]

| **Table S1.** Average measurement of morphometric traits, across seasonal and geographic climatic gradients, in specimens from the present survey. (a) *Orius albidipennis* and (b) *O. niger*. | | | | | |
| --- | --- | --- | --- | --- | --- |
|  | Region | Month | Wing length (mm) | Thorax width (mm) | Wing to thorax size ratio |
|  |  |  | Average ± SE | Average ± SE | Average ± SE |
| **(a)^1^** | Mediterranean plain | Jan (n = 23) | 1.50 ± 0.012 | 0.66 ± 0.005 | 2.26 ± 0.010 |
|  |  | Apr (n = 88) | 1.56 ± 0.016 | 0.71 ± 0.008 | 2.21 ± 0.014 |
|  |  | Aug (n = 73) | 1.24 ± 0.009 | 0.58 ± 0.004 | 2.13 ± 0.007 |
|  |  | Nov (n = 135) | 1.43 ± 0.008 | 0.64 ± 0.004 | 2.23 ± 0.005 |
|  | Semi- arid | Jan (n = 27) | 1.58 ± 0.013 | 0.69 ± 0.006 | 2.29 ± 0.009 |
|  |  | Apr (n = 199) | 1.52 ± 0.013 | 0.70 ± 0.005 | 2.17 ± 0.009 |
|  |  | Aug (n = 57) | 1.22 ± 0.004 | 0.57 ± 0.002 | 2.14 ± 0.004 |
|  |  | Nov (n = 250) | 1.37 ± 0.005 | 0.62 ± 0.002 | 2.22 ± 0.004 |
| **(b)** | Mountainous Mediterranean | Jan (n = 13) | 1.63 ± 0.026 | 0.75 ± 0.011 | 2.18 ± 0.016 |
|  |  | Apr (n = 104) | 1.63 ± 0.006 | 0.76 ± 0.003 | 2.15 ± 0.005 |
|  |  | Aug (n = 9) | 1.29 ± 0.015 | 0.61 ± 0.011 | 2.14 ± 0.021 |
|  |  | Nov (n = 43) | 1.52 ± 0.012 | 0.70 ± 0.005 | 2.17 ± 0.007 |
|  | Mediterranean plain | Jan (n = 78) | 1.62 ± 0.009 | 0.75 ± 0.005 | 2.17 ± 0.012 |
|  |  | Apr (n = 213) | 1.59 ± 0.004 | 0.74 ± 0.002 | 2.14 ± 0.004 |
|  |  | Aug (n = 45) | 1.30 ± 0.008 | 0.62 ± 0.005 | 2.10 ± 0.011 |
|  |  | Nov (n = 20) | 1.54 ± 0.017 | 0.72 ± 0.007 | 2.14 ± 0.008 |
|  | Semi- arid^1^ | Jan (n = 19) | 1.65 ± 0.023 | 0.76 ± 0.011 | 2.16 ± 0.010 |
|  |  | Apr (n = 19) | 1.57 ± 0.015 | 0.74 ± 0.008 | 2.12 ± 0.011 |
| ^1^ Data for *O. albidipennis* in the Mountainous Mediterranean region and for *O. niger* in August and November in the Semi-arid region are not included due to small sample sizes. | | | | | |
